# Nutzen maximieren – Schaden minimieren: Neugeborenen-Screening in Deutschland

**DOI:** 10.1007/s00103-023-03784-3

**Published:** 2023-09-27

**Authors:** Inken Brockow, Joseph Kuhn, Uta Nennstiel

**Affiliations:** Bayerisches Landesamt für Gesundheit und Lebensmittelsicherheit, Veterinärstr. 2, 85764 Oberschleißheim, Deutschland

Früherkennungsuntersuchungen und speziell bevölkerungsbezogene Screening-Programme spielen für die präventive Orientierung im Gesundheitswesen eine – auch mit Blick auf die Fortschritte diagnostischer Verfahren – immer wichtigere Rolle. Konstitutiv für Screening-Programme sind definierte Zielkrankheiten und Zielgruppen, ein systematisches Einladungssystem, die standardisierte Aufklärung über Nutzen und Risiken, vorhandene Behandlungsansätze sowie die Qualitätssicherung einschließlich einer Prozess- und Ergebnisevaluation. Die bekanntesten Screening-Programme in Deutschland sind vermutlich die Früherkennung von Brustkrebs (seit 2009), Darmkrebs (seit 2019) und Gebärmutterhalskrebs (seit 2020). Ihre Grundlage ist das Krebsfrüherkennungs- und -registergesetz (KFRG; siehe dazu auch Heft 12/2018 des Bundesgesundheitsblatts).

Das Neugeborenen-Screening aus Trockenblutkarten (NBS) ist in Deutschland bereits Ende der 1960er-Jahre mit Einführung des Screenings auf Phenylketonurie (PKU) etabliert worden und mittlerweile eine der effektivsten Maßnahmen der Sekundärprävention. Seit Einführung des NBS konnten in Deutschland mehr als 15.000 Kinder mit einer der Zielkrankheiten durch das Screening frühzeitig entdeckt und einer Behandlung zugeführt werden. Dadurch war es möglich, schwere Krankheitsfolgen bis hin zu Behinderung und Tod zu vermeiden oder zumindest zu mildern. Allerdings kann ein Screening auch schaden, z. B. durch Ressourcenverbrauch im Gesundheitssystem und unnötige Beunruhigung der Eltern bei falsch-positiven Befunden. Es gilt, durch die Etablierung eines Screening-Programms den Nutzen zu maximieren und möglichen Schaden zu minimieren.

Da bei diesem Bevölkerungs-Screening die gesamte Population vorwiegend gesunder Neugeborener ohne das Vorliegen klinischer Symptome oder eines erhöhten Risikos auf bestimmte Erkrankungen untersucht wird, sind an die Prozessqualität im analytischen sowie prä- und postanalytischen Screening-Ablauf besonders hohe Anforderungen zu stellen. Während die Durchführung des NBS in Deutschland in der Kinder-Richtlinie des Gemeinsamen Bundesausschusses (G-BA) geregelt ist, fehlen in diesen Regelungen wichtige Punkte eines Screening-Programms, wie z. B. die Sicherstellung der Vollständigkeit und ein umfassendes Qualitätsmanagement. Der erste Beitrag zum Leitthema von *Nennstiel et al.* gibt hier wichtige Empfehlungen zur Weiterentwicklung des deutschen NBS, die im Rahmen eines Konzepts im Auftrag des Spitzenverbands der gesetzlichen Krankenkassen (GKV-Spitzenverbandes) erarbeitet wurden. Als Beispiel für ein herausragendes zentral koordiniertes Screening-Programm mit einer guten Homepage für die Eltern wird von *Schulze und Chakraborty* das NBS in Ontario, Kanada skizziert.

Einen Überblick über die Analytik des NBS im Laufe der Zeit gibt der Artikel von *Janzen und Sander.* Neue technische Möglichkeiten bei der Analytik, insbesondere die Etablierung der Tandem-Massenspektrometrie 1999, bei der in einem Arbeitsschritt verschiedene Parameter erfasst werden können, führten zu einer Erweiterung der Zielkrankheiten. So wurde das im Jahr 2004 sogenannte erweiterte Neugeborenen-Screening auf 10 Stoffwechselkrankheiten und 2 Hormonstörungen in die Kinder-Richtlinie und damit in den Leistungskatalog der GKV aufgenommen und im Laufe der letzten Jahre um 5 Krankheiten aus unterschiedlichen pädiatrischen Fachgebieten erweitert. Die Herausforderungen bei der Einführung einer neuen Zielkrankheit werden in diesem Themenheft am Beispiel des im Jahr 2019 eingeführten Screenings auf schwere angeborene Immundefekte (SCID) von *Ghosh et al.* beschrieben.

Im Zusammenhang mit Kindern, die selbst nicht einwilligungsfähig sind, stellen sich ethische Grundprobleme der Früherkennung von Krankheiten, z. B. was wünschenswertes Wissen, das Recht auf Nichtwissen oder eine sensible Befundmitteilung angeht, in besonderer Weise. Das Neugeborenen-Screening kann in dieser Hinsicht als „Labor des Umgangs mit Wissen“ gesehen werden, dessen Diskussionsergebnisse weit über das Neugeborenen-Screening selbst hinausreichen. Dies gilt insbesondere für die Einführung molekulargenetischer Analysen für ein populationsbasiertes genomisches Neugeborenen-Screening. Die Anwendung von Hochdurchsatz-Sequenziermethoden bietet zahlreiche Chancen für die Verbesserung der Bevölkerungsgesundheit, aber auch zahlreiche Herausforderungen und noch offene Fragen bei ethischen, rechtlichen, sozialen, psychologischen und technischen Aspekten, die im Artikel von *Brennenstuhl und Schaaf *beleuchtet werden.

Für die Qualitätssicherung eines Screenings ist eine regelmäßige Evaluation des gesamten Screening-Prozesses notwendig. Voraussetzung dafür ist eine standardisierte Dokumentation aller Screening-Parameter, inkl. der Ergebnisse der Konfirmationsdiagnostik, nach Möglichkeit in Registern. Möglichkeiten und Grenzen bei der Erfassung dieser Daten in unterschiedlichen Quellen werden im Beitrag von *Nährlich und Brockow* diskutiert. Nur durch die Erfassung des Langzeit-Outcomes der betroffenen Kinder können wichtige Fragen, z. B. bisher unklare natürliche Verläufe und die phänotypische Vielfalt seltener Krankheiten, verstanden werden. Auch die unzureichende Möglichkeit, den Schweregrad und Verlauf der Krankheit für einzelne Individuen vorherzusagen, Unsicherheiten bei der Falldefinition zu reduzieren sowie eine Risikostratifizierung der Behandlungsindikation nach der Diagnosestellung vorzunehmen, kann nur durch die Auswertung des Langzeitverlaufes der betroffenen Kinder verbessert werden. Einen Überblick dazu gibt der Artikel von *Mütze und Kölker* zu den Ergebnissen der Langzeit-Untersuchung von Kindern mit Stoffwechselstörungen in Heidelberg.

Im Jahr 2009 wurde bundesweit auch ein universelles Neugeborenen-Hörscreening als Kassenleistung durch Aufnahme in die Kinder-Richtlinie eingeführt. Die Umsetzung des Neugeborenen-Hörscreenings in Deutschland wurde durch eine Bietergemeinschaft im Auftrag des G‑BA für die Jahre 2011/2012 und erneut 2017/2018 evaluiert. Die Ergebnisse werden im Beitrag von *Brockow et al*. zusammengefasst.

Dieses Heft soll einen Überblick über die bisherige Entwicklung, Ergebnisse und Herausforderungen des Neugeborenen-Screenings in Deutschland liefern und Anregungen für notwendige programmatische Weiterentwicklungen zur weiteren Optimierung des Screenings geben.

Aus dem Neugeborenen-Screening lassen sich auch Schlussfolgerungen ableiten für andere, auch nicht laborgestützte Früherkennungsuntersuchungen bei Kindern, für den sich abzeichnenden Einsatz von künstlicher Intelligenz bei Früherkennungsuntersuchungen sowie für die Einbettung medizinischer Untersuchungen in übergreifende Versorgungskonzepte, wie sie z. B. beim nationalen Gesundheitsziel „Gesundheit rund um die Geburt“ verfolgt werden.

Den Autorinnen und Autoren danken wir für ihre Beiträge und hoffen, mit diesem Themenheft die Diskussion zum Neugeborenen-Screening in Deutschland um neue Perspektiven zu bereichern.

